# Collagenous Gastritis: An Atypical Presentation of a Rare Disease

**DOI:** 10.7759/cureus.34698

**Published:** 2023-02-06

**Authors:** Jaclyn E Kagihara, Julia L Boland, Giancarlo Colon Rosa, Divya Mamilla, Mamoun Younes, Marie L Borum, Samuel A Schueler

**Affiliations:** 1 Division of Gastroenterology and Liver Diseases, George Washington University School of Medicine and Health Sciences, Washington, DC, USA; 2 Internal Medicine, George Washington University School of Medicine and Health Sciences, Washington, DC, USA; 3 Pathology, George Washington University School of Medicine and Health Sciences, Washington, DC, USA

**Keywords:** anemia, iron deficiency, esophagogastroduodenoscopy, collagen deposition, egd, iron deficiency anemia (ida), gastritis, collagenous gastritis

## Abstract

Collagenous gastritis is a rare inflammatory condition of unknown etiology defined histologically by subepithelial deposition of collagen bands ≥ 10 µm in the lamina propria. Adults typically present with diarrhea, often attributed to concurrent collagenous sprue or collagenous colitis. Children more commonly present with abdominal pain and anemia, with inflammation typically limited to the stomach. Herein, we present a case of collagenous gastritis in a 38-year-old female with a history of iron deficiency and hypothalamic amenorrhea who presented with a one-year history of microcytic anemia. Celiac disease panel, *Helicobacter pylori* testing, and anti-parietal cell and intrinsic factor antibodies were negative. Esophagogastroduodenoscopy revealed diffusely erythematous and nodular gastric mucosa in the antrum and pylorus. Biopsy from the gastric body showed complete loss of oxyntic glands and deposition of a thick band of collagen under the surface epithelium infiltrated by a few eosinophils, consistent with collagenous gastritis with severe atrophy. She was treated with omeprazole 40 mg daily for six weeks and iron supplementation. Our patient’s symptoms and endoscopic findings are consistent with previously described pediatric, but not adult, cases of collagenous gastritis, yielding insight into the variable clinical presentation of this rare disease.

## Introduction

Collagenous gastritis (CG) is a rare condition marked by inflammation, which drives subepithelial deposition of collagen bands in the stomach [1]. The prevalence of CG on esophagogastroduodenoscopy (EGD) is approximately 13 per 100,000 EGDs [2]. Limited data on this condition have demonstrated a bi-modal age distribution, with pediatric patients typically presenting with anemia and abdominal pain [3]. Adults typically present with diarrhea and sometimes present with concomitant collagenous colitis or other autoimmune diseases [2]. Herein, we report an atypical presentation of CG in an adult female who presented with iron deficiency anemia and abdominal pain.

This article was previously presented as a meeting poster at the 2021 American College of Gastroenterology (ACG) Annual Scientific Meeting on October 22, 2021.

## Case presentation

A 38-year-old woman with a six-year history of iron deficiency and hypothalamic amenorrhea presented to our clinic with a one-year history of anemia. The evaluation showed a hemoglobin of 9.9 grams/deciliter (g/dl) (baseline: 13-14 g/dL), iron deficiency with a ferritin level of 4 nanograms/milliliter (ng/ml), and vitamin B12 deficiency with a level of 144 picograms (pg)/ml (Table 1). She endorsed pagophagia. She was referred to gastroenterology given concerns for malabsorption as the cause of her iron and vitamin B12 deficiency.

**Table 1:** Initial laboratory values Abbreviations: g: grams; dL: deciliter; MCV: mean corpuscular volume; fL: femtoliters; mg: milligrams; ng: nanograms; U: units; Ig: immunoglobulin.

Lab	Value	Reference range
Hemoglobin (g/dL)	9.9	11.1-15.9
MCV (fL)	75	79-97
Iron saturation (%)	7	15-55
Iron (mg/dL)	33	27-159
Ferritin (ng/mL)	4	15-150
Immunoglobulin A, quantitative (mg/dL)	96	87-352
Deamidated gliadin antibodies, IgA (U)	4	0-19
Deamidated gliadin antibodies, IgG (U)	9	0-19
T-transglutaminase (tTG) IgA (U/mL)	<2	0-3
T-transglutaminase (tTG) IgG (U/mL)	3	0-5

In the gastroenterology clinic, she noted intermittent mild periumbilical cramping but denied melena or hematochezia. She denied fevers, chills, weight loss, dysphagia, or diarrhea. She had no history of non-steroidal anti-inflammatory drugs or alcohol use. She had a copper intrauterine device and was not on any medications. She had no notable family history. Vital signs were normal. A physical exam was normal. Notable negative labs included *Helicobacter pylori*, anti-parietal cell and intrinsic factor antibodies, and a celiac panel. A complete metabolic panel, fat-soluble vitamins, and immunoglobulin levels were normal.

**Figure 1:**
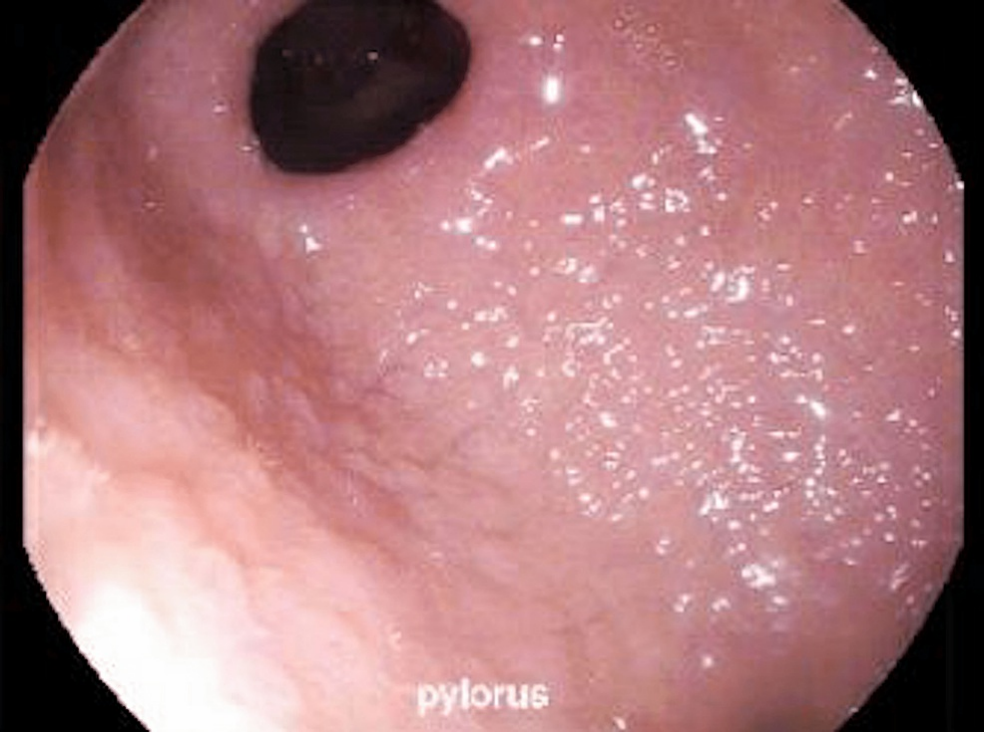
Diffusely nodular gastric mucosa seen in the pre-pyloric region on esophagogastroduodenoscopy

**Figure 2:**
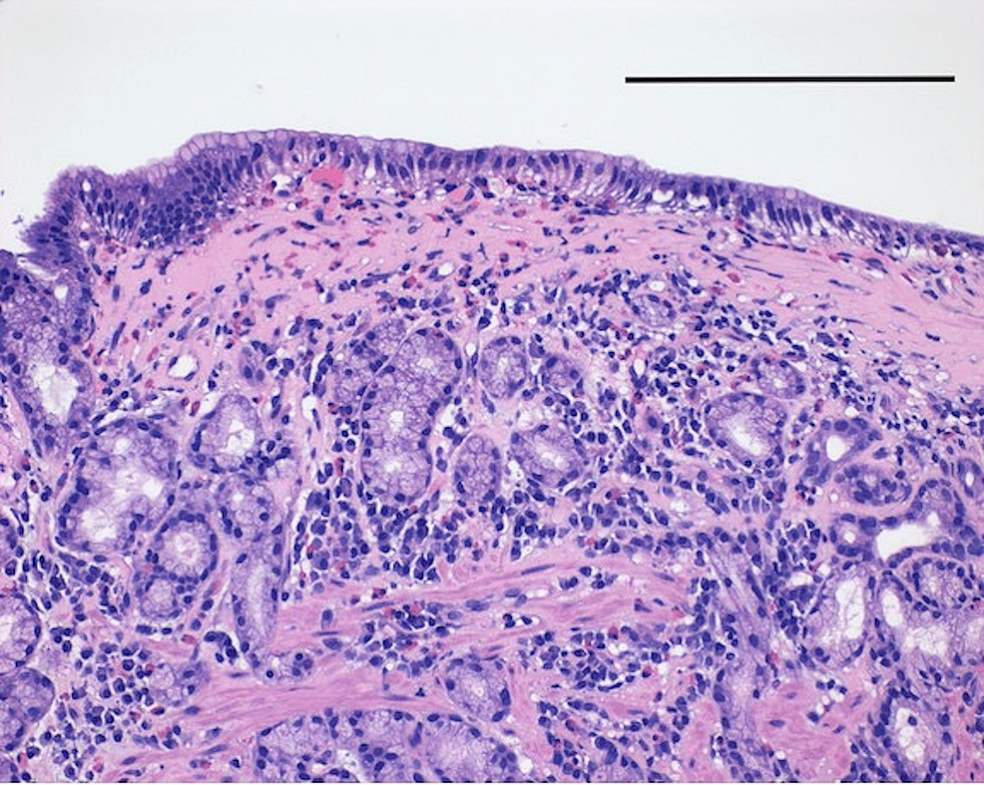
Hematoxylin and eosin staining of gastric body biopsy, 20x microscope objective A biopsy from the gastric body shows a complete loss of oxyntic glands and deposition of a thick band of collagen under the surface epithelium infiltrated by a few eosinophils. Scale bar = 0.25 millimeters.

EGD revealed diffusely nodular and erythematous gastric mucosa (Figure 1). Random gastric biopsies demonstrated marked friability of the gastric mucosa with subsequent bleeding resulting in famotidine 40 milligrams (mg) intravenously given intra-procedurally. Pathology revealed CG with deposition of a thick band of collagen under the surface epithelium infiltrated by a few eosinophils, and the absence of oxyntic glands and parietal cells (Figure 2). *Helicobacter pylori* testing was negative. Colonoscopy with intubation of the terminal ileum was unremarkable.

The patient was treated with omeprazole 40 mg by mouth once a day for six weeks. In the interim, she was started on vitamin B12 supplementation. At the two-month follow-up, she noted occasional sporadic abdominal cramping but was otherwise well. Repeat hemoglobin was 10.1 g/dl, and ferritin was 5 ng/mL. She was started on ferrous sulfate 325 mg by mouth once a day.

## Discussion

CG is a rare inflammatory condition of unknown etiology defined histologically by subepithelial deposition of collagen bands ≥ 10 µm in the lamina propria. Theories of the pathogenesis of CG include inflammation and autoimmune injury [4]. It is hypothesized that subepithelial fibroblasts release collagen in response to chronic inflammation [3]. CG has been associated with lymphatic disorders of the GI tract such as lymphocytic gastritis, celiac disease, and duodenal intraepithelial lymphocytosis [2].

Adults with CG typically present with diarrhea, often attributed to concurrent collagenous sprue or collagenous colitis, and may have coexisting autoimmune diseases [2]. Children more commonly present with abdominal pain and anemia, with inflammation typically limited to the stomach [5]. The etiology of anemia in CG may be associated with the friability of the gastric lining from dilated capillaries entrapped in the collagen bands [3]. Endoscopic findings characteristically include nodularity in the gastric body; however, there are variable findings [1]. Some reported cases of CG suggest obtaining biopsies from gastric mucosa with cobblestone appearance may be of higher diagnostic yield [6].

Three distinct inflammatory patterns are appreciated: a lymphocytic gastritis-like pattern, an eosinophil-rich pattern, and an atrophic gastritis pattern [7]. There are no standard therapies or surveillance recommendations, and long-term significance, including malignant potential in the setting of chronic inflammation, remains unclear. Reports of this diagnosis have shown the condition to relapse and persist for years in some cases [4,8]. All patients reported with CG were treated with acid suppression, either a proton pump inhibitor or an H2 blocker, as in our case. Some patients reported in the literature were treated with courses of steroids, with little to no improvement [3]. A recent study utilized topically targeted budesonide (TTB) for the treatment of CG, with 89% demonstrating a clinical response and 88% demonstrating a histologic response to TTB [9].

Our patient’s symptoms and endoscopic findings are consistent with previously described pediatric, but not adult, cases of CG, yielding insight into the variable clinical presentation of this rare disease. A recent review found just 60 cases of CG reported from 1989 to 2015, highlighting the importance of further reports to inform the approach toward recognition, management, and surveillance [10].

## Conclusions

CG is rare, and its pathogenesis is unknown. There is a female predominance, and it commonly manifests as abdominal pain and anemia in pediatric patients. This case demonstrates an atypical presentation of CG in an adult patient with iron deficiency anemia. Typical pediatric patient endoscopy reveals a nodular appearing stomach and iron deficiency anemia hypothesized to result from bleeding superficial capillaries entrapped in collagen. Adults tend to present more commonly with chronic watery diarrhea attributed to a strong association with collagenous colitis. To date, there is no standard therapy, and the long-term significance of this disease is unclear. Further studies are warranted to elucidate the pathogenesis and long-term course of this disease.
